# Development and Content Validation of a Transcultural Instrument to Assess Organizational Readiness for Knowledge Translation in Healthcare Organizations: The OR4KT

**DOI:** 10.15171/ijhpm.2018.17

**Published:** 2018-03-06

**Authors:** Marie-Pierre Gagnon, Randa Attieh, Sandra Dunn, Gonzalo Grandes, Paola Bully, Carole A. Estabrooks, France Légaré, Geneviève Roch, Mathieu Ouimet

**Affiliations:** ^1^Population Health and Optimal Health Practices Research Unit, CHU de Québec-Université Laval Research Centre, Québec, QC, Canada.; ^2^Faculty of Nursing, Université Laval, Québec, QC, Canada.; ^3^CRED Research Centre – École Supérieure des Affaires, Beirut, Lebanon.; ^4^CHEO Research Institute, Centre for Practice Changing Research Building, Ottawa, ON, Canada.; ^5^Better Outcomes Registry & Nerwork (BORN) Ontario, Ottawa, ON, Canada.; ^6^Primary Care Research Unit of Bizkaia – Osakidetza, Basque Health Service, Bilbao, Spain.; ^7^BioCruces Health Research Institute, Baracaldo, Spain.; ^8^Faculty of Nursing and School of Public Health, University of Alberta, Edmonton, AB, Canada.; ^9^Department of Family Medicine, Université Laval, Québec, QC, Canada.; ^10^Department of Nursing Sciences, Université Laval, Québec, QC, Canada.

**Keywords:** Healthcare Organizations, Readiness for Change, Knowledge Translation, Instrument Development, Transcultural Validation

## Abstract

**Background:** Implementing effective interventions in healthcare requires organizations to be ready to support change. This study aimed to develop, adapt transculturally, and assess the content and face validity of the Organizational Readiness for Knowledge Translation (OR4KT) tool. The OR4KT was designed to measure the readiness of healthcare organizations to implement evidence-informed change across a variety of services.

**Methods:** Based on systematic reviews of the literature, a Delphi exercise, and expert consultation, we first generated an initial pool of items. Second, we developed and assessed content validity of the pilot OR4KT questionnaire in English. Third, we created French and Spanish versions using a sequential forward and backward translation approach, and transcultural adaptation by a consensus process. Finally, we conducted pilot studies in three contexts – the Basque country region (Spain), and the provinces of Québec and Ontario (Canada) – where 30 experts assessed the face validity of the three versions of OR4KT.

**Results:** We selected 59 items, grouped in 6 dimensions (organizational climate, context, change content, leadership, organizational support, and motivation) for the final English version of OR4KT. Translation and transcultural adaptation did not identify any content or language problems. Our findings indicate that the English, French and Spanish versions of OR4KT are linguistically equivalents and have high face validity. Only minor revisions to the wording of some items were recommended.

**Conclusion:** The OR4KT holds promise as a measure of readiness for knowledge translation (KT) in healthcare organizations. The validity and reliability of the three versions of the OR4KT will be assessed in real-life contexts of implementation of evidence-based changes in healthcare

## Background

Healthcare organizations need to be ready to adapt to constantly changing demands and environments.^1^ Organizational readiness (OR) constitutes an important concept to operationalize in order to assess organizational capacity to engage in implementing evidence-informed change in healthcare.^1,2^

The implementation of evidence-informed practice, conceptualized as the integration of scientific research, patients’ preferences, professional expertise and available resources,^3^ is promoted to improve care. However, several studies highlight the difficulties of translating evidence to the ‘real-life’ care context.^4-6^ According to the literature, high levels of OR contribute to successful change in healthcare^2^ and business organizations.^7,8^ Therefore, organizations aiming to implement evidence-informed change require OR for knowledge translation (KT).^1,9,10^ KT is a complex endeavor, thus preparatory work is needed to enhance implementation outcomes.^11^

Although OR is recognized as a potential facilitator of effective KT, there is currently a lack of consensus regarding how to assess it.^12^ Instruments specifically designed to assess OR for KT in healthcare organizations or existing instruments that could be used for this purpose are lacking.^2,13-15^ Indeed, in a previous systematic review^1^ we identified a limited number of valid and reliable measurements that could be readily used in healthcare settings to assess the degree of readiness to implement evidence-informed change. Another limitation of current OR tools is that they have been developed for research purpose and could be burdensome for use by busy clinicians and healthcare managers.^16^ However, the findings of our systematic review lay groundwork for the development of a comprehensive instrument based upon available frameworks to assess OR for KT to support implementation of evidence-informed practices.^1^ Hence, we developed an instrument that can help to assess the readiness of healthcare organizations to implement evidence-informed change.

The purpose of this article is to describe the development and validation of the OR4KT, a comprehensive instrument for assessing the readiness of healthcare organizations to implement evidence-informed change in healthcare organizations. This instrument was developed based on two systematic reviews: one of OR theoretical components,^1^ and one of OR measurement tools.^17^ The OR4KT is designed to help decision makers to assess capacity within an organization or across a set of organizations to implement evidence-informed change.^18^

## Methods

The OR4KT instrument was created in three phases: initial development of the pilot OR4KT, content validity and item reduction, transcultural adaptation and face validity assessment (see Figure).

**Figure:**
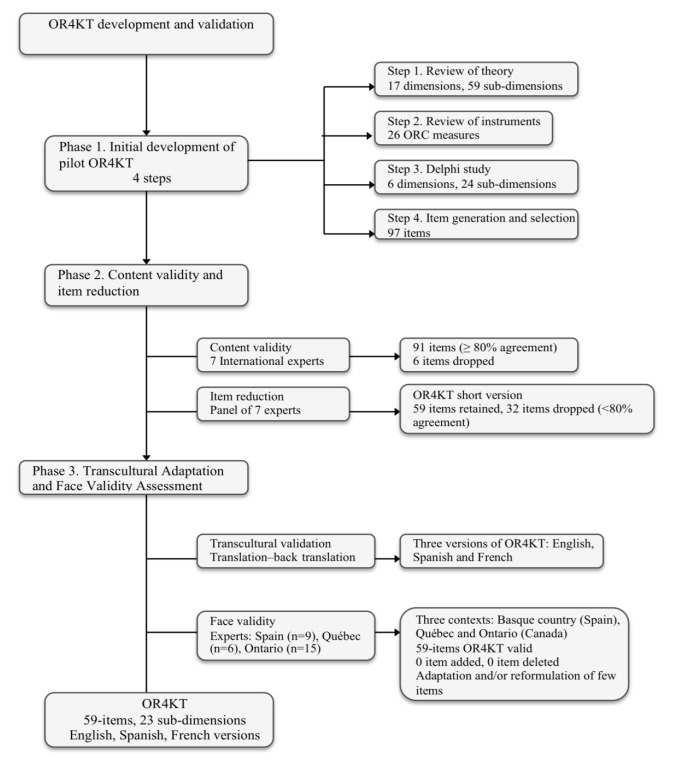
Flowchart of the Development and Validation of the Organizational Readiness for Knowledge Translation (OR4KT) Measurement Instrument.

### Phase 1: Initial Development of Pilot OR4KT

The initial version of OR4KT instrument was developed in four steps including (1) review of theory, (2) review of instruments, (3) Delphi study and expert panel, and (4) item generation and reduction.

#### Step 1 – Review of Theory

Initially, the research team conducted an extensive review of conceptual models/frameworks of OR for change^19^ in healthcare at the organizational level.^1^ The focus was to understand OR components relevant to KT interventions. This preliminary work led to the development of a conceptual map of the different components of OR gathered from the retained 10 theories, theoretical models and conceptual frameworks. First, three of the authors (GR, MPG, and RA) placed the elements extracted from the frameworks and models identified in the included publications using the CmapTools software. Concepts were represented in a hierarchical fashion with the most inclusive, most general concepts at the top of the map and the more specific, less general concepts at the bottom. Then, in a brainstorming session, the three authors identified which elements were at the highest theorization level (concepts). From the remaining elements, they distinguished dimensions and sub-dimensions, which represented second or third level theorization. Third, the three authors sought relationships among the concepts, dimensions and sub-dimensions that were created. Fourth, they placed related dimensions and sub-dimensions near each other within the concept to which they related in the concept map.

From this conceptual map, we identified five core concepts that have been used to operationalize OR for KT (organizational dynamics, change process, innovation readiness, institutional readiness, and personal readiness).^1^

#### Step 2 – Review of Instruments

The existing valid OR measurement instruments that could be applied to KT in the healthcare sector were systematically reviewed by the same research team.^20^ Twenty-six valid and/or reliable OR measures described in 39 publications^20^ were identified through a systematic review as relevant for measuring OR for KT in the healthcare domain at the organizational level. These measures were examined for their components and provided 17 dimensions and 59 sub-dimensions in relation to the identified five core concepts.

#### Step 3 – Delphi Exercise and Expert Consultation

We then conducted a web-based, double-round, modified Delphi exercise.^12^ A panel of 10 international experts in the fields of OR or KT rated their agreement concerning the importance and applicability of OR components. The aim of the Delphi was to reduce the number of dimensions and sub-dimensions identified from the systematic review and ensure their relevance to the field of OR for KT in healthcare. After two rounds, experts reached consensus on a total of 6 dimensions and 28 sub-dimensions. Given the small change in results after the second round of the Delphi exercise, we consulted a panel of seven international experts in the fields of OR or KT from our contacts who were not involved in the Delphi exercise. Based on the comments/suggestions received, four sub-dimensions were eliminated and their items were eliminated or relocated to other sub-dimensions, leaving a total of 24 sub-dimensions.

#### Step 4 – Item Generation and Selection

On the basis of the Delphi exercise and expert consultation, we selected items that best matched the retained dimensions and sub-dimensions. Based on the pool of items from original OR instruments and our proposed items, we designed a preliminary version of the OR4KT, comprising 97 items grouped into six dimensions and 24 sub-dimensions.

### Phase 2: Content Validation and Item Reduction

The purpose of this phase was to assess the content and face validity of the OR4KT questionnaire and proceed to item reduction.

### Content Validation

In order to assess the relevance of each selected item in measuring the related sub-dimension retained in the Delphi exercise, we invited seven international experts in the fields of KT or OR measurement to participate in the study. We asked the experts to indicate whether or not each of the proposed items were relevant to assess the related sub-dimension of the OR4KT questionnaire and/or to revise items if needed. Following that process, 91 items with high agreement (≥80%) were retained.

### Item Reduction

After presenting the original 91-items version of the OR4KT for application in the context of a study in the Basque country region, we proceeded to reduce items given that the original tool was considered too long for use by primary healthcare organizations. A panel of seven experts, including two physician investigators, one psychologist, one nurse, one manager, one researcher and one graduate student, were emailed an invitation letter soliciting their participation in the item reduction process. They were asked to identify, based on their experience, which items could be removed within each dimension without affecting the overall coherence of the scale. After considering experts’ suggestions on which items could be removed, the research team produced a short version of the OR4KT instrument containing 59 items across 23 sub-dimensions.

### Phase 3: Transcultural Adaptation and Face Validity Assessment

The reduced version of the OR4KT instrument consisting of 59 items originally developed in English was translated by experienced translators into Spanish and French. A native Spanish-speaker and a native French-speaker initially translated the original OR4KT English version into Spanish and French. Careful attention was given to the readability and clarity of the items. Linguistic equivalence across the various language versions was achieved by translation–back translation process and avoidance of jargon, idioms and metaphors.^21^ First, the translated Spanish and French versions were reviewed jointly between translators and bilingual researchers fluent either in Spanish and/or French regarding ambiguities and discrepancies of words, sentences and meanings. In the next step, a back-translation (Spanish to English and French to English) was carried out in order to assure the quality of translation between source and target languages.^22^ The OR4KT Spanish and French versions were back translated to English by two independent native English-speakers who are fluent in Spanish and French. These translators were not the same people who participated in the original translation and they were not aware of the existing English version. Single iteration was performed for most of the items for the two target versions. The original English version and the two retranslated versions were compared and evaluated for their linguistic equivalence by the research team. Items found to be discrepant between the original English version and the retranslated ones were discussed until consensus was achieved about revisions required. The final versions of the OR4KT instrument produced in this phase in English, Spanish and French were used in the validation process.

### Face Validity of Transculturally Adapted OR4KT

After we developed the 59-items French and Spanish versions of OR4KT, we conducted a pilot study in three contexts including the Basque country region of Spain, and the provinces of Québec and Ontario in Canada, aiming to judge anonymously the items on face validity. The purposes of this phase were to assess the clarity of the wording of the items in the initial item pool and to generate new items from experts if needed. Thirty experts from Basque country, Québec and Ontario were invited to participate in the face validity process of the OR4KT Spanish, French and English versions. These experts were representatives of healthcare providers involved in KT projects, healthcare managers and researchers/methodologists.

The Spanish version of OR4KT was adapted to the context of changes in preventive practices in primary healthcare organizations in Basque country. The instrument was sent to a panel of nine experts involving two physicians, three nurses, two researchers and two managers. In Québec, the OR4KT was adapted to the context of electronic patient health portal (ePHP) implementation in primary healthcare organizations. Six experts including two physicians, two nurses and two administrative managers were invited to determine face validity per item of French OR4KT instrument.

In Ontario, fifteen experts, including four researchers, eight clinicians (with medical, nursing and midwifery background), and three analysts (with biostatistical and epidemiological expertise), participated in three review rounds to validate the tool. The English OR4KT version was adapted for use in a provincial survey evaluating an electronic audit and feedback system to support quality improvement in the context of maternal and newborn care in Ontario.

All invited experts were asked to make remarks or comments on the plausibility and comprehensiveness of the items, textual shortcomings, omissions, redundancies, clarity of the wording of the items, relevance of the five-point Likert scale (ranging from 1: strongly disagree to 5: strongly agree) and the questionnaire length.

## Results

### Item Reduction

After item reduction, we produced a final version of the OR4KT measurement instrument containing 59 items across 6 dimensions and 23 sub-dimensions (Supplementary file 1). Thus, 32 items with less than 80% agreement were rejected. All items were assessed on a 5-point Likert scale, from strongly disagree to strongly agree.

### Translation Process

Translation of the OR4KT tool into French and Spanish, and back translation into English did not result in any major content or language problems. All questions were worded precisely and comprehensibly. Our findings demonstrated linguistic equivalence between the original (English) and target languages (French and Spanish) of the OR4KT. The final French translated instrument is shown in Supplementary files 2, and the Spanish translated instrument is available in an article published by Grandes et al.^23^

### Validity

Face validity of the three versions of the OR4KT instrument was assessed by experts from Spain (n = 9), Québec (n = 6), and Ontario (n =15). The majority of respondents completed the questionnaire in 15 to 20 minutes. Although respondents highlighted some items that were difficult to understand (see below), they did not suggest deleting or adding any item to the final OR4KT version.

In the English version, experts identified a number of issues about questions that were unclear or confusing. They suggested rewording several items in order to add clarity to the item pool (Table). A definition of some terms has also been added for clarity. For instance, the term ‘innovation decision maker’ is defined as ‘a person in your organization who can facilitate decision-making about whether to adopt a new way of doing something to improve healthcare delivery.’

**Table:** Selected Examples of Problematic Items in the Three Versions of OR4KT Questionnaire

	**Original Items**	**New Reformulated/Phrased Items**
Original English OR4KT version	Item 11: determines classification of roles and responsibilities in relation to specific change application	Considers roles and responsibilities as part of the planning process in relation to specific change initiatives
	Item 48: the evaluation and improvement of the change implementation include review of results by leadership	Leaders review results to evaluate and improve the planned changes
	Item 54: funding organizations make pressures for change	Pressure to make change comes from funding organizations
Translated OR4KT French version	Item 26: les changements proposés ont été bien acceptés par les patients	Les changements proposés ont généralement été bien acceptés par les patients
	Item 32: les gestionnaires demandent aux professionnels d’obtenir des résultats	Les gestionnaires responsabilisent les professionnels en vue de l’atteinte des résultats
	Item 47: l’évaluation et l’amélioration de la mise en œuvre des changements comprennent un plan de diffusion des mesures de performance	Les mesures de performance sont communiquées aux parties prenantes afin d’évaluer et d’améliorer la mise en œuvre des changements
Translated OR4KT Spanish version	Item 2: los profesionales están habitualmente pendientes y se ayudan entre si cuando se necesita	Los profesionales están habitualmente pendientes de ayudarse entre si cuando se necesita
	Item 9: los gestores están abiertos a las ideas de los profesionales para propiciar los cambios	La dirección de comarca está abierta a las ideas de los profesionales para propiciar los cambios
	Item 27: los cambios propuestos se basan en las necesidades y preferencias de los pacientes	Los cambios propuestos toman en consideración las necesidades y preferencias de los pacientes

Abbreviation: OR4KT, Organizational Readiness for Knowledge Translation.

In the Spanish version, eight items were reformulated (Table). For instance, ‘employee’ was changed to ‘professional,’ and ‘managers’ was replaced by ‘health district authority (*dirección de comarca*).’ Experts emphasized the importance to refer to a specific health center or health region when asking about management, because that would help responses to the survey to be more practical and easier.

For the French version, experts also emphasized the need to add specifications and/or to reformulate items. Four items were reformulated (Table). Wording specifications were performed for three items retained. Experts highlighted the need to define what the terms ‘external stakeholders,’ ‘course of change,’ and ‘innovation decision-maker’ meant. Revisions were incorporate to address this concern. Thus, the same definitions provided in the English OR4KT version were also added to the French and Spanish versions in order to ensure equivalence of the three versions of the OR4KT instrument.

Finally, all experts agreed that the length of the questionnaire (that took about 15 minutes to complete) and the proposed 5-point agreement scale were appropriate.

## Discussion

The aim of this study was to develop and validate the OR4KT instrument to gauge OR for KT in healthcare organizations. The OR4KT instrument consists of 59 items grouped under six dimensions, to assess an organization’s predisposition to support implementation of evidence-informed practices. We developed three linguistically equivalent versions of the OR4KT (English, Spanish, and French) that provide a valid, specific and easily administered measure of readiness in healthcare organizations to implement evidence-informed change. Face validity of each version was guaranteed by an international panel of experts including clinicians, managers, and researchers in three distinct linguistic and cultural settings: the Basque county region (Spain), and the provinces of Ontario and Québec (Canada). The experts suggested making minor changes in the wording and/or phrasing of a few items, and adding definitions to clarify some concepts.

Our findings indicated that the English, French, and Spanish versions of the 59-items OR4KT instrument are clearly understood. The French and Spanish versions showed a linguistic equivalence to the original English version, based on the translation/back translation process.

Face and content validity are qualitative measures that are regarded as important first steps in the instrument development. Face and content validity assessed the appearance, relevance and representativeness of the OR4KT instrument. After establishing face and content validity, other types of validity need to be considered in the OR4KT validation process. To ensure a psychometrically sound instrument, the next steps consist of assessing the instrument’s criterion-related validity either concurrent or predictive validity and construct validity in the three different contexts implementing evidence-based changes in healthcare. The Spanish version of the OR4KT has already been validated in the context of the implementation of chronic disease prevention in primary healthcare organizations of the Basque Health Service.^23^ Further research is needed to investigate the potential of the OR4KT as a measure of OR for KT in a variety of contexts and applications and its predictive capability for healthcare practice change to implement proven interventions.

## Strengths and Limitations

One strength of this study lies in the development and transcultural validation of the OR4KT in three different linguistic, cultural, and clinical settings. Another strength is the ability of the OR4KT instrument to measure readiness to change from different dimensions regarded as important in healthcare organizations. The OR4KT is a general assessment instrument which could be applied in a variety of healthcare organizations preparing for the implementation of evidence-informed practices. With minor adaptations, the questionnaire could also be used to support other changes and/or interventions in the healthcare sphere.

However, this study also presents limitations due to the specific contexts in which the OR4KT tool was developed. Indeed, to ensure further validation of the tool, the transcultural adaptation and face validation processes would need to be replicated in other contexts. We hypothesize that the theoretical foundations on which the OR4KT is based provide enough support for its application in other contexts, but the specific items that were developed in this study could not apply in specific settings, such as low- and middle-income countries, for instance. Thus, it is important to pursue the development and testing of the OR4KT. Another limitation of this study is the limited evidence on validity of the instrument. Face validity is commonly considered a minimum validation requirement.^24^ Questions are reviewed to see if they seem to relate to the measured attribute. To ensure the development of a valid and reliable instrument, the validation process of OR4KT still needs to be supplemented by other types of validity. The assessment of item reliability, and construct and criterion validity of OR4KT questionnaire has been conducted in Basque country^23^ and is currently undergoing in Québec and Ontario. Next steps could be conducting an invariance analysis between the three versions of the questionnaire and prospective evaluation of its predictive capability for successful implementation.

## Conclusion

This study led to the development, transcultural adaptation and face validity evaluation of the OR4KT, a unique instrument that can be applied to gauge healthcare organizations’ readiness to implement evidence-based practices. Our results show that the transcultural validation process has led to the development of French and Spanish versions of the OR4KT instrument that are equivalent to the original English version. The three versions of the OR4KT also show good face validity, and minor rewording was done following suggestions from experts. In conclusion, the OR4KT holds promise as a measure of readiness for KT in healthcare organizations.

## Acknowledgements

We want to thank Professor Jeremy Grimshaw, principal investigator of the KT Canada project, for his essential support to the realization of this work. We acknowledge the contribution of all the experts who participated in either the Delphi study, the item reduction panel, the face and content validation of the OR4KT. In particular, we want to thank the teams of clinicians and investigators in Ontario, Quebec, and Euskadi who took their precious time to comment the initial questionnaire versions. The OR4KT instrument would not exist without their input and relevant comments. We also thank the translators for their much-appreciated contribution.

## Ethical issues

Ethical approval for the study was obtained from the research ethics committee of the Hospital St-François d’Assise of the CHU de Quebec (approved on March 13, 2013; ethics number B13-04-657). Experts participating in this study were informed that their participation was entirely voluntary and they were free to withdraw at any time. By responding to the questionnaire, they implicitly consented to participate in the study. The study was anonymous and no personal information was collected.

## Competing interests

Authors declare that they have no competing interests.

## Authors’ contributions

MPG, FL, CE, and MO conceived the idea and obtained funding. RA and MPG conducted the design, development and validation of the questionnaire. GG, PB, SD, MPG, and RA participated in the process of the questionnaire validation. RA and MPG wrote the first draft of the manuscript with significant input from GG, PB, and SD. All authors commented and contributed to the final manuscript.

## Funding

This project is supported by a team grant operated by Knowledge Translation Canada and offered by the Canadian Institutes of Health Research (CIHR), Ottawa, ON, Canada in partnership with the Canada Foundation for Innovation (CFI), Ottawa, ON, Canada (grant # 200710CRI-179929-CRI-ADYP-112841). MPG is Tier 2 Canada Research Chair in Technologies and Practices in Health. FL is Tier 1 Canada Research Chair in Shared Decision Making and Knowledge Translation.

## Authors’ affiliations

^1^Population Health and Optimal Health Practices Research Unit, CHU de Québec-Université Laval Research Centre, Québec, QC, Canada. ^2^Faculty of Nursing, Université Laval, Québec, QC, Canada. ^3^CRED Research Centre – École Supérieure des Affaires, Beirut, Lebanon. ^4^CHEO Research Institute, Centre for Practice Changing Research Building, Ottawa, ON, Canada. ^5^Better Outcomes Registry & Nerwork (BORN) Ontario, Ottawa, ON, Canada. ^6^Primary Care Research Unit of Bizkaia – Osakidetza, Basque Health Service, Bilbao, Spain. ^7^BioCruces Health Research Institute, Baracaldo, Spain. ^8^Faculty of Nursing and School of Public Health, University of Alberta, Edmonton, AB, Canada. ^9^Department of Family Medicine, Université Laval, Québec, QC, Canada. ^10^Department of Nursing Sciences, Université Laval, Québec, QC, Canada.

## Supplementary files

Supplementary file 1: 59-items OR4KT English version.

Supplementary file 2: 59-items OR4KT French version.

## Key messages

**Implications for policy makers**

- Policy-makers are in strong need to assess the organizational context and its needs prior to implement evidence-informed practices.
- The Organizational Readiness for Knowledge Translation (OR4KT) questionnaire is tailored to measure the readiness of healthcare organizations to implement evidence-informed change across a variety of services.
- The OR4KT was developed and validated in three languages: French, English and Spanish, extending the scope of its use in different social and cultural contexts.
- The OR4KT could provide support to policy-makers when implementing evidence-informed change in healthcare organizations.
- With the availability of few valid and reliable measures, the OR4KT is promising as a measure of readiness for evidence-informed change in healthcare organizations.

**Implications for the public**

The healthcare system needs to provide a ready environment for implementing change informed by scientific evidence. Therefore, it is essential to assess whether organizations have the necessary resources, competencies, skills, support, motivation, and information in order to ensure successful change implementation and to improve care. To do so, the Organizational Readiness for Knowledge Translation (OR4KT) measurement instrument has been developed and validated in three different languages, English, French and Spanish. Our study shows that the OR4KT is an acceptable and valid instrument for exploring the context of healthcare organizations when implementing evidence-informed change. This tool can be used in a variety of healthcare organizations preparing for the implementation of practices informed by scientific knowledge.

## References

[1] Attieh R, Gagnon MP, Estabrooks CA (2013). Organizational readiness for knowledge translation in chronic care: a review of theoretical components. Implement Sci.

[2] Weiner BJ, Amick H, Lee SY (2008). Conceptualization and measurement of organizational readiness for change: a review of the literature in health services research and other fields. Med Care Res Rev.

[3] Yost J, Ganann R, Thompson D (2015). The effectiveness of knowledge translation interventions for promoting evidence-informed decision-making among nurses in tertiary care: a systematic review and meta-analysis. Implement Sci.

[4] Montague TJ, Gogovor A, Krelenbaum M (2007). Time for chronic disease care and management. Can J Cardiol.

[5] Grimshaw JM, Eccles MP, Lavis JN, Hill SJ, Squires JE (2012). Knowledge translation of research findings. Implement Sci.

[6] Brown CE, Wickline MA, Ecoff L, Glaser D (2009). Nursing practice, knowledge, attitudes and perceived barriers to evidence-based practice at an academic medical center. J Adv Nurs.

[7] Kotter JP. Leading Change. Boston: Harvard Business Press; 1996.

[8] Armenakis AA, Harris SG, Mossholder KW (1993). Creating Readiness for Organizational Change. Hum Relat.

[9] Scott SD, Estabrooks CA, Allen M, Pollock C (2008). A context of uncertainty: how context shapes nurses’ research utilization behaviors. Qual Health Res.

[10] Cummings GG, Estabrooks CA, Midodzi WK, Wallin L, Hayduk L (2007). Influence of organizational characteristics and context on research utilization. Nurs Res.

[11] Graham ID, Logan J, Harrison MB (2006). Lost in knowledge translation: time for a map?. J Contin Educ Health Prof.

[12] Attieh R, Gagnon MP, Estabrooks CA (2014). Organizational readiness for knowledge translation in chronic care: a Delphi study. BMC Health Serv Res.

[13] Ehrhart MG, Aarons GA, Farahnak LR (2014). Assessing the organizational context for EBP implementation: the development and validity testing of the Implementation Climate Scale (ICS). Implement Sci.

[14] Holt DT, Helfrich CD, Hall CG, Weiner BJ (2010). Are you ready? How health professionals can comprehensively conceptualize readiness for change. J Gen Intern Med.

[15] Timmings C, Khan S, Moore JE, Marquez C, Pyka K, Straus SE (2016). Ready, Set, Change! Development and usability testing of an online readiness for change decision support tool for healthcare organizations. BMC Med Inform Decis Mak.

[16] Shea CM, Jacobs SR, Esserman DA, Bruce K, Weiner BJ (2014). Organizational readiness for implementing change: a psychometric assessment of a new measure. Implement Sci.

[17] Gagnon MP, Attieh R, Ghandour el K (2014). A systematic review of instruments to assess organizational readiness for knowledge translation in health care. PLoS One.

[18] Gagnon MP, Labarthe J, Legare F (2011). Measuring organizational readiness for knowledge translation in chronic care. Implement Sci.

[19] Higgins R, Murphy B, Worcester M, Daffey A (2012). Supporting chronic disease self-management: translating policies and principles into clinical practice. Aust J Prim Health.

[20] Bowditch JL, Buono AF. A Primer on Organizational Behavior. 5th ed. New York: John Wiley Sons, Limited; 2002.

[21] Touw-Otten F, Meadows K. Cross-cultural issues in outcome measurements. In: Hutchinson A, McColl E, Christie M, Riccalton CS, eds. Health Outcome Measures in Primary and Out-Patient Care. Amsterdam: Hardwood Academic Publishers; 1996:199-208.

[22] Maneesriwongul W, Dixon JK (2004). Instrument translation process: a methods review. J Adv Nurs.

[23] Grandes G, Bully P, Martinez C, Gagnon MP (2017). Validity and reliability of the Spanish version of the Organizational Readiness for Knowledge Translation (OR4KT) questionnaire. Implement Sci.

[24] Streiner DL, Norman GR. Health Measurement Scales: A Practical Guide to Their Development and Use. 4th ed. Oxford (UK): Oxford University Press; 2008.

