# Supplementary material for: Development and Content Validation of a Transcultural Instrument to Assess Organizational Readiness for Knowledge Translation in Healthcare Organizations: The OR4KT
Source: Int J Health Policy Manag. 2018 Mar 6;7(9):791–7. doi: 10.15171/ijhpm.2018.17 (PMC6186488; doi:10.15171/ijhpm.2018.17)
Supplement: Supplementary file 1 — 59-items OR4KT English version. [file ijhpm-7-791-s001.pdf]

Organizational Readiness for Knowledge Translation

(OR4KT) Questionnaire

On a five-point Likert scale, please assess the degree to which the following statements correspond to your organization:

1 = Stongly disagree; 2 = Disagree; 3 = Neutral; 4 = Agree; 5 = Strongly agree

1. Organizational climate for change

In your organization:	1	2	3	4	5
1- staff work together as a team.					
2- staff are usually quick to help one another when needed.					
3- mutual trust among staff is strong.					
4- the heavy workload reduces intervention effectiveness.					
5- staff frustration is common.					
6- ideas and suggestions from staff get fair consideration by senior leaders.					
7- the formal communication channels work very well.					
8- staff members feel free to ask questions and express concerns.					
9- managers are open to staff ideas for improving change.					
10- managers encourage trying new and different practices.					

2- Organizational contextual factors

Your organization:	1	2	3	4	5
1- determines classification of roles and responsibilities in relation to specific change application(s).					
2- has necessary support in terms of budget or financial resources.					
3- has necessary support in terms of training.					
4- has necessary support in terms of facilities and equipment.					
5- has necessary support in terms of staffing numbers.					
In your organization:					
6- managers solicit opinions of clinical staff regarding decisions about patient care.					
7- staff members have a sense of personal responsibility for improving patient care and outcomes.					
8- staff members cooperate to maintain and improve effectiveness of patient care.					
9- staff members are willing to innovate and/or experiment to improve clinical procedures.					
10- staff members are receptive to change in clinical processes.					

3- Change content

In your organization:	1	2	3	4	5
1- there is willingness to adjust to changes.					
2- there is ability to exchange ideas and impact upon decisions related to delivery of patient care.					
3- there is flexibility to deal with change.					

4- people are willing to adjust usual routines in response to what is happening around them.					
5- typically there is ability to adapt new standards or procedures, even those forced upon us.					
6- the proposed changes have been well accepted by patients in a pilot study.					
7- the proposed changes take into consideration the needs and preferences of patients.					
8- the proposed changes appear to have more advantages than disadvantages for patients.					
9- the proposed changes should be effective, based on current scientific knowledge.					

4- Leadership

In your organization:	1	2	3	4	5
1- managers provide effective management for continuous improvement of patient care.					
2- managers provide staff members with feedback/data on effects of clinical decisions.					
3- managers hold staff members accountable for achieving results.					
4- external stakeholders are involved in the planning process.					
5- all the staff members are usually included in decision-making processes.					
6- there is an innovation decision maker on key organizational clinical committees.					
7- there is an innovation decision maker on key organizational administrative committees.					
8- managers are involved in change process.					
9- clinicians are involved in the change process.					
10- administrative and clerical staff are involved in the change process.					

5- Organizational support

In your organization:	1	2	3	4	5
1- team members provide practical support for new ideas and their application.					
2- assistance in developing new ideas is readily available.					
3- team members cooperate in order to help develop and apply new ideas.					
4- team members provide and share resources to help in the application of new ideas.					
5- the change progress is monitored continuously.					
6- outcomes are monitored continuously.					
7- the evaluation and improvement of the change implementation include periodic outcome measurement.					
8- the evaluation and improvement of the change implementation include dissemination plan for performance measures.					
9- the evaluation and improvement of the change implementation include review of results by leadership.					
10- there is formal mechanism established for obtaining feedback related to the proposed change.					

6- Motivation

In your organization:	1	2	3	4	5
1- patients make pressures to make changes.					
2- pressures to make changes come from staff members.					
3- senior leaders make pressures to make changes.					

4- pressures to make changes come from board members or overseers.					
5- funding organizations make pressures for change.					
6- there is implementation change experience gained from projects or pilot programs and their evaluation.					
7- managers are knowledgeable about innovation based on their past experience					
8- knowledge is available about how similar innovations are being used by other organizations.					
9- senior managers promote change by behaving in a manner consistent with it.					
10- senior managers define the course of change.					

Comments:

____
____
____
____
____
____
____

Thank you for your participation!
