# Supplementary material for: Development and Content Validation of a Transcultural Instrument to Assess Organizational Readiness for Knowledge Translation in Healthcare Organizations: The OR4KT
Source: Int J Health Policy Manag. 2018 Mar 6;7(9):791–7. doi: 10.15171/ijhpm.2018.17 (PMC6186488; doi:10.15171/ijhpm.2018.17)
Supplement: Supplementary file 2 — 59-items OR4KT French version. [file ijhpm-7-791-s002.pdf]

PREPARATION ORGANISATIONNELLE A L'APPLICATION DES CONNAISSANCES DANS LES SOINS DE SANTE
QUESTIONNAIRE OR4KT

Le questionnaire OR4KT vise à recueillir des informations sur certains aspects organisationnels de votre organisation, en particulier par rapport aux changements qui pourraient survenir dans les pratiques professionnelles en lien avec l'utilisation d'un portail électronique par les patients pour la gestion des maladies chroniques.

Ce questionnaire est composé de 59 questions et le temps estimé par l'équipe de recherche pour le compléter est estimé à 15 minutes.

Toutes les informations que vous nous fournissez seront traitées de façon strictement confidentielle et ne seront en aucun cas utilisées individuellement, mais feront partie de l'analyse globale de toutes les enquêtes recueillies.

Merci beaucoup pour votre collaboration!

Sur une échelle Likert de cinq points, veuillez s.v.p. évaluer votre unité de soins de santé primaires selon les énoncés suivants :

1 = Fortement en désaccord; 2 = En désaccord; 3 = Neutre; 4 = En accord; 5 = Fortement en accord

**1. Climat organisationnel pour le changement**					
Dans votre organisation:	**1**	**2**	**3**	**4**	**5**
1- les professionnels travaillent en équipe					
2- les professionnels s'entraident en cas de besoin					
3- la confiance mutuelle est forte entre les professionnels					
4- la surcharge de travail réduit le succès de l'intervention*					
5- Les professionnels vivent souvent des frustrations					
6- les idées et les suggestions des professionnels reçoivent l'attention voulue par les gestionnaires					
7- les voies de communication officielles fonctionnent très bien					
8- les professionnels se sentent libres de poser des questions et exprimer leurs préoccupations					
9- les gestionnaires sont ouverts aux idées des professionnels pour promouvoir le changement					
10- les gestionnaires encouragent les pratiques nouvelles et différentes					
**2- Facteurs contextuels organisationnels**					
Votre organisation:	**1**	**2**	**3**	**4**	**5**
11- détermine la classification des rôles et des responsabilités en ce qui concerne la mise en œuvre d'un changement spécifique					
12- a le soutien nécessaire en termes de ressources budgétaires et économiques					

13- a le soutien nécessaire en termes de formation					
14- a le soutien nécessaire en termes d'installations et d'équipements					
15- a le soutien nécessaire en termes de personnel					
16- les gestionnaires sollicitent l'opinion des cliniciens à prendre des décisions sur les soins aux patients					
17- les professionnels ont un sens de responsabilité personnelle pour améliorer les soins aux patients et leur impact sur la santé de ces derniers					
18- les professionnels coopèrent afin d'améliorer et de maintenir l'efficacité des soins aux patients					
19- les professionnels sont prêts à innover et / ou expérimenter pour améliorer les procédures cliniques					
20- les professionnels sont réceptifs au changement dans les processus cliniques					
**3- Contenu du changement**					
Dans votre organisation:	**1**	**2**	**3**	**4**	**5**
21- il y a la volonté de s'adapter aux changements					
22- il y a la capacité d'échanger des idées et avoir un impact sur les décisions relatives à la prise en charge du patient					
23- il y a la flexibilité pour faire face aux changements					
24- les gens sont prêts à apporter des ajustements aux routines habituelles en réponse à ce qui se passe autour d'eux					
25- généralement, les nouvelles règles ou procédures peuvent être adaptées au contexte, y compris celles qui ont été imposées					
26- les changements proposés ont été bien acceptés par les patients					

27- les changements proposés tiennent compte des besoins et préférences des patients					
28- les changements proposés semblent avoir plus d'avantages que d'inconvénients pour les patients					
29- les changements proposés doivent être efficaces, basés sur les connaissances scientifiques actuelles					
**4- Leadership**					
Dans votre organisation:	**1**	**2**	**3**	**4**	**5**
30- les gestionnaires fournissent une gestion efficace de l'amélioration continue des soins aux patients					
31- les gestionnaires offrent aux professionnels des informations/données sur les effets des décisions cliniques					
32- les gestionnaires demandent aux professionnels d'obtenir des résultats					
33- les parties prenantes externes à votre organisation s'impliquent dans le processus de planification					
34- généralement, tous les professionnels sont inclus dans les processus décisionnels					
35- il y a un décideur de l'innovation dans les comités cliniques					
36- il y a un décideur de l'innovation dans les comités administratifs					
37- les gestionnaires s'impliquent dans le processus de changement					
38- les cliniciens s'impliquent dans le processus de changement					
39- le personnel administratif et bureautique s'implique dans le processus de changement					
**5- Support Organisationnel**					
Dans votre organisation:	**1**	**2**	**3**	**4**	**5**

40- les membres soutiennent les nouvelles idées et leur application					
41- de l'aide est facilement disponible pour développer de nouvelles idées					
42- les membres de l'équipe collaborent pour aider à développer et à mettre en œuvre de nouvelles idées					
43- les membres de l'équipe fournissent et partagent des ressources pour aider à la mise en œuvre de nouvelles idées					
44- le processus de changement est surveillé en permanence					
45- les résultats sont surveillés en permanence					
46- l'évaluation et l'amélioration de la mise en œuvre du changement comprennent des mesures périodiques des résultats					
47- l'évaluation et l'amélioration de la mise en œuvre des changements comprennent un plan de diffusion des mesures de performance					
48- l'évaluation et l'amélioration de la mise en œuvre des changements comprennent un examen des résultats par les dirigeants					
49- il y a un mécanisme formel en place pour recueillir des commentaires relatifs aux modifications proposées					
**6- Motivation**					
Dans votre organisation:	**1**	**2**	**3**	**4**	**5**
50- les patients font pression pour faire des changements					
51- les pressions pour le changement proviennent des employés					
52- les gestionnaires poussent au changement					
53- les pressions pour le changement proviennent du conseil d'administration ou des superviseurs					

54- les organismes subventionnaires poussent au changement					
55- il existe une expérience précédente de projets ou de programmes pilotes ayant nécessité une gestion du changement					
56- les gestionnaires sont bien informés sur l'innovation grâce à leurs expériences antérieures					
57- il y a des informations disponibles sur l'utilisation d'innovations similaires par d'autres organisations					
58- Les gestionnaires favorisent le changement en se comportant en conformité avec ce changement					
59- les gestionnaires planifient le déroulement du changement					

Commentaires:

___

___

___

___

___

___

___

___

___

___

___

Merci pour votre participation!
